# Fine-tuning characterization of patients with interstitial pneumonia and an underlying autoimmune disease in real-world practice: We get closer with Nailfold videocapillaroscopy

**DOI:** 10.3389/fmed.2023.1057643

**Published:** 2023-02-15

**Authors:** Fredeswinda Isabel Romero-Bueno, Maria Jesús Rodríguez-Nieto, Carmelo Palacios Miras, Lina Martínez Estupiñán, Maria José Martínez-Becerra, Maria Carmen Vegas Sánchez, Oderay Mabel Cedeño Díaz, Olga Sánchez-Pernaute, The NEREA Autoimmune ILD Study Group

**Affiliations:** ^1^Rheumatology Department, IIS-HU Fundación Jiménez Díaz, Autonoma University, Madrid, Spain; ^2^Department of Pulmonology, IIS-HU Fundación Jiménez Díaz, Autonoma University and CIBERES, Madrid, Spain; ^3^Department of Imaging, IIS-HU Fundación Jiménez Díaz, Autonoma University, Madrid, Spain; ^4^Department of Immunology, IIS-HU Fundación Jiménez Díaz, Autonoma University, Madrid, Spain; ^5^Department of Pathology, IIS-HU Fundación Jiménez Díaz, Autonoma University, Madrid, Spain

**Keywords:** connective tissue diseases (CTD), interstitial pneumonia (iP), nailfold videocapillaroscopy, interstitial pneumonia with autoimmune features (IPAF), anti synthetase syndrome

## Abstract

**Objectives:**

To assess performance of interstitial pneumonia (IP) with autoimmune features (IPAF) criteria in clinical practice and describe the utility of additional workup in identifying patients with underlying connective tissue diseases (CTD).

**Methods:**

We set a retrospective study of our patients with autoimmune IP, who were allocated to CTD-IP, IPAF or undifferentiated autoimmune IP (uAIP) subgroups according to the updated classification criteria. Presence of the process-related variables comprising IPAF defining domains was scrutinized in all patients, and, when available, the results of nailfold videocapillaroscopy (NVC) were recorded.

**Results:**

Thirty nine out of 118 patients, accounting for 71% of former undifferentiated cases, fulfilled IPAF criteria. Arthritis and Raynaud’s phenomenon were prevalent in this subgroup. While systemic sclerosis-specific autoantibodies were restricted to CTD-IP patients, anti-tRNA synthetase antibodies were also present in IPAF. In contrast, rheumatoid factor, anti-Ro antibodies and ANA nucleolar patterns could be found in all subgroups. Usual interstitial pneumonia (UIP) / possible UIP were the most frequently observed radiographic patterns Therefore, the presence of thoracic multicompartimental findings as also performance of open lung biopsies were useful in characterizing as IPAF those UIP cases lacking a clinical domain. Interestingly, we could observe NVC abnormalities in 54% of IPAF and 36% of uAIP tested patients, even though many of them did not report Raynaud’s phenomenon.

**Conclusion:**

Besides application of IPAF criteria, distribution of IPAF defining variables along with NVC exams help identify more homogeneous phenotypic subgroups of autoimmune IP of potential relevance beyond clinical diagnosis.

## Introduction

Interstitial lung diseases (ILD) or interstitial pneumonia (IP) are a group of conditions characterized by chronic damage at the lung parenchyma resulting from an abnormal remodeling and sustained inflammatory response upon an initial injury of the alveolar and/or microvascular structure. While sharing clinical, radiological and functional characteristics, ILD show a wide range of presentations and a variable risk of functional deterioration and fibrosis development. Their heterogeneity relies not only on different etiologic backgrounds but also on host-dependent factors, which account for a variety of perpetuating mechanisms (1). In order to improve the standard of care of these patients it is fundamental to correctly characterize individual phenotypes both as regards to the underlying disease and to the participation of targetable processes.

The investigation of an underlying condition in patients with ILD can be challenging. After an exhaustive workup aimed to rule out distinct etiologic factors, such as drugs and other environmental toxics, infectious agents, allergens, genetic disorders or some neoplasms, most patients can be classified into any of the following diagnostic groups: autoimmune connective tissue disease (CTD) associated IP, granulomatous processes and idiopathic IP (IIP), of which idiopathic pulmonary fibrosis (IPF) is the most characteristic form (2). Although the IIP diagnosis accounts for approximately 15% of ILD cases, the group of CTD-IP is expanding as a result of recent advances in the characterization of autoantibodies as well as in CTD-classification criteria. It is expected that this tendency will continue to grow in the next few years while, in parallel, the number of IIP forms will go down, since most of the patients could have an autoimmune substrate (3, 4). This fact is anticipated, due to the unfavorable prognosis of IPF as compared to CTD-IP (5).

In clinical practice, there is a substantial overlap between CTD-IP and IIP suggesting a continuum in the pathogenesis of chronic fibrotic IP processes (6). In 2015, a joint European Respiratory Society (ERS) - American Thoracic Society (ATS) Task Force on undifferentiated forms of CTD-IP developed preliminary criteria for the classification of patients as Interstitial Pneumonia with Autoimmune Features (IPAF) in order to identify patients with high suspicion of an underlying autoimmune process but not fulfilling criteria for a definite CTD (7).

Nailfold videocapillaroscopy (NVC) is an accesible and non-invasive image technique which allows the direct exam of microvessels morphology. This examination is principally performed in patients with Raynaud’s phenomenon -or in those ones with a clinical suspicion of systemic sclerosis (SSc)-to confirm the presence of SSc typical vasculopathy (8). However, microvascular involvement may be associated to additional CTDs, such as dermatomyositis and the anti-aminoacyl tRNA synthetase syndrome (ARS). It should be underlined that these entities do not necessarily present with Raynaud’s phenomenon. Subsequently, NVC is being increasingly used by rheumatologists in the assessment of patients with subtle or overlapping CTD features. The latter include patients with IP, since this manifestation may reflect organic involvement of SSc and related CTDs. In this regard, capillary loss at nailfolds has been shown to associate with IP in patients with SSc, but NVC abnormalities have also been found in patients diagnosed with other CTD-IP, IPAF or even in IPF (9). At present, in spite of its routinary use in Multidisciplinary IP Clinics, the precise utility of this examination in the accurate classification of patients with IIP remains to be established (9).

In this study, we have reviewed the cohort of patients attending our Multidisciplinary Clinic for autoimmune ILD in order to find out the prevalence of both IPAF and IPAF-defining items as well as the presence of abnormalities in NVC searching for a better characterization of the patients.

## Methods

### Study population

The study was conducted in accordance with the declaration of Helsinki and was approved by Jimenez Diaz Foundation University Hospital Ethics Committee. Data were collected by retrospective chart review. The study population comprised patients attending the Jimenez Diaz Foundation University Hospital multidisciplinary autoimmune ILD Clinic between January 2011 and January 2020. Patients were not directly involved in their participation. Presently, all patients with active follow-up at our Clinic are offered participation in a multicenter prospective register of patients with autoimmune IP of Madrid (NEREA register). The authors will disseminate the study results to the NEREA cohort of patients. We will provide a short summary of our principal findings at the NEREA web site and send a hard copy to the participating centers to be available at the multidisciplinary ILD clinics.

### Inclusion criteria and subgroup definitions

In order to be included in the analysis, patients needed have a clinical diagnosis of ILD in the context of an autoimmune disease, after ruling out alternative etiologies (10). In all cases, the final diagnosis had been confirmed by a multidisciplinary team with the participation of Radiologists, Pathologists, Rheumatologists and Pulmonologists. Only patients with at least 3-year follow-up from diagnosis or a fatal outcome were included. Based on the updated classification criteria for the different CTD (8, 11–15) and on Fischer’s IPAF criteria (7), patients were re-classified by the investigators. Those of them not fulfilling any of these criteria were grouped as undifferentiated autoimmune interstitial pneumonia (uAIP). Besides, patients were diagnosed with anti-aminoacyl tRNA synthetase syndrome (ARS) following Solomon’s preliminary criteria (16).

### Additional data in patients’ characterization

Along with demographics, all relevant data for the classification of the disease, which included the assessment of the IPAF domain-defining variables, were listed. In those patients who had undergone a diagnostic surgical lung biopsy, the histopathological findings were registered.

### Nailfold videocapillaroscopy

When available, the result and characteristics of NVC performed as per clinical practice during diagnostic workup were also recorded. All nailfold videocapillaroscopy (NVC) studies had been performed according to an internal protocol which complies with EULAR recently launched recommendations (17). Briefly, patients are instructed to avoid smoking, cafeine or manicure before the examination and a short period of acclimatizationto room temperatura is set. Studies performed before 2014 were done with a Zuzi® Optical stereomicroscope (optical magnification of 50x) and an adapted Optikam® camera or with an USB Digital Microscope video epiluminiscence Dino-Lite® (200x magnification). A Nikon SMZ-745 T stereomicroscope with 6.7x - 50x zoom range equipped with a led stand, white epi-illuminators and an 12 MP (pixel size 1.85 micrometer) resolution USB camera (DFK 33UX226, The Imaging Source) is used from 2014 onwards. Image capturing and processing is done with The Imaging Source software. For an improved image acquisition, the nailfolds are bathed with a thin layer of immersion oil (Sigma-Aldrich). Acquisition and interpretation are performed by trained rheumatologists.All images are automatically coded and stored. Supplementary File 1 shows definition of NVC abnormalities as applied in this study along with an illustration of the standardized evaluation of lesions (17, 18). For the purpose of this study, all the images were reviewed by the same blinded examiner.

### Statistical analysis

Descriptive statistics are shown as frequencies (%) or mean (SEM) and median. Differences in categorical variables were analyzed with Pearson’s Chi2 test or Fischer’s exact test, while quantitative measures were compared with independent two sample T test or ANOVA followed by Dunnett’s t test when applicable. Associations are shown as odds ratio with CI95 or as positive and negative predictive values where applies. A 2-sided *p* value of 0.05 was considered significant.

## Results

### Cohort characteristics

One hundred and eighteen patients, whose characteristics are shown in Table 1, were included. Most of the patients in our cohort were Spanish, followed by a 10% of Hispanics. There was a predominance of women (69% of the patients). Median disease duration was 8 years and total follow-up was 984 patient-years (PY). Thirty one disease-related deaths occurred during this period (3.1/100PY). According to current classification criteria, 53% of the patients (*n* = 63) had a definite CTD, while an additional 33% (*n* = 39) fulfilled IPAF criteria and 14% (*n* = 16) remained unclassified. There was a marked difference between diagnostic subgroups as regards to age at onset, being patients diagnosed with a CTD 9.15 ± 3.3 years younger than those with IPAF (*p* 0.017), and 11.8 ± 4.5 than those of the uAIP group (p 0.026). Comorbidities did not substantially differ between groups. Table 2 summarizes demographics of the CTD-IP subgroup, which mostly comprised patients with rheumatoid arthritis (RA, *n* = 22), inflammatory myopathies (IM, *n* = 21) and systemic sclerosis (SSc, *n* = 16). As it is shown in the Table, some patients met classification criteria of 2 different CTDs. On the other hand, there were 21 patients in the cohort fulfilling criteria for ARS, whose characteristics can be found in Supplementary File 2. Of note, according to current classification criteria, 15 (71%) of the latter patients were included in the CTD-IP diagnostic group, while 6 (29%) of them could not be classified as a definite CTD but fulfilled IPAF criteria instead. Supplementary File 3 provides an overview of the considerable overlapping between conditions evidenced in our study population.

**Table 1:** Study population. All patients were diagnosed with autoimmune interstitial pneumonia.

	Total cohort (*n* = 118)	Clinical subgroups			Multigroup comparisons	Two-group comparisons difference: mean ± SEM [CI95]		
		CTD-IP (*n* = 63)	IPAF (*n* = 39)	uAIP (*n* = 16)		CTD-IP vs. IPAF	CTD-IP vs. uAIP	IPAF vs. uAIP
Women, *n* (%)	82 (69)	48 (76)	26 (67)	8 (50)	ns			
Hispanic, *n* (%)	12 (10)	9	2	1	ns			
Age at onset, mean (SEM), *median*	59 (1), *62*	55 (2), *56*	64 (3), *66*	66 (34), *70*	*p* 0.004	dif: 9.1 ± 3.3 [1.3; 16.8] p 0.017	dif: 11.8 ± 4.5 [1.2; 22.4] p 0.026	ns
Age at endpoints, mean (SEM), *median*	70 (1), *72*	68 (2), *68*	73 (2), *75*	75 (2), *77*	*p* 0.07	ns	ns	ns
Disease duration (year), mean (SEM), *median*	8.3 (0.5), *8*	8.4 (0.7), *8*	8.9 (1), *8*	6.9 (1), *6.5*	ns	ns	ns	ns
Follow-up, patient-years	984	527	347	110				
First manifestation								
pulmonary, *n* (%)	45 (38)	12 (19)	22 (56)	11 (69)	*p* < 0.001	*p* < 0.001	*p* 0.001	ns
Extra-pulmonary, *n* (%)	44 (37)	35 (56)	7 (18)	2 (13)				
Synchronic, *n* (%)	29 (25)	16 (25)	10 (26)	3 (19)				
Smoking exposure								
Never	53 (45)	34 (54)	13 (33)	6 (37)	*p* 0.024	ns	*p* 0.021	ns
Past	43 (36)	16 (25)	17 (44)	10 (62)				
Active	20 (17)	12 (19)	8 (20)	0				
SPY, mean (SEM), *median*	31.5 (3.9)	30 (7) *16*	35 (6) *35*	27 (8) *20*	ns			
Comorbidities at onset, *n* (%)	59 (50)	36 (57)	16 (44)	10 (62)	ns			
Past or present history of cancer, *n* (%)	20 (17)	12 (19)	7 (18)	1 (6)	ns			
Organ-specific autoimmune condition, *n* (%)	34 (29)	17 (27)	9 (23)	8 (50)	ns			
Deaths, *n* (%)	31 (26)	14 (22)	10 (26)	7 (43)	ns			

Patients are allocated to three clinical subgroups according to fulfillment of classification criteria for a definite connective tissue disease (CTD-IP), interstitial pneumonia with autoimmune features (IPAF) or none (uAIP) at the end of follow-up.

**Table 2:** CTD-IP cases. Patients are allocated according to fulfillment of classification criteria for the different connective tissue diseases (CTD) at the end of follow-up.

	Rheumatoidarthritis	Systemicsclerosis	Inflammatorymyopathy	Sjögren	Lupus	Mixedconnectivetissuedisease
Number	21 + 1 overlapping case	14 + 2 ovarlapping cases	20 + 1 overlapping case	4 + 1 overlapping case	1 ovarlapping case	1
Women, *n* (%)	16 (73)	12 (75)	15 (75)	4 (100)	1 (100)	1 (100)
Eversmoker, *n* (%)	10 (48)	4 (29)	14 (67)			
Smoking status, *n*: never, past, active smoker	12, 5, 5	12, 4, 0	7, 7, 7	4, 0, 0	1, 0, 0	1, 0, 0
Age at onset, mean (SEM) *median*	60.1 (3.4) *62*	46.8 (4.5) *48*	51.8 (2.4) *53.5*			
Age at endpoints, mean (SEM) *median*	76.3 (2.4) *80*	61.4 (3.9) *63*	60.8 (2) *61.5*			
Disease duration, mean (SEM) *median*	9.1 (1.5) *8*	9.1 (1.3) *8.5*	6.8 (1.1) *6.5*			
Follow-up, (patient-years)	200	146	139	53	9	3
Respiratory onset, *n*	4	3	3	2	0	0
Extra-respiratory onset, *n*	18	10	5	2	1	1
Synchronic pulmonary and extra pulmonary disease at onset, *n*	0	3	13	1	0	0

As regards to disease onset, 38% of the patients presented with an isolated pulmonary condition, including 12 patients (19%) of the CTD subgroup. Notwithstanding, most of the latter could be classified by the Rheumatologist during diagnostic workup. There were only 3 patients who changed diagnosis later on due to the appearance of new manifestations. These were 1 patient initially classified as uAIP who developed definite RA within the first year of symptoms, and 2 additional patients presenting with IPAF who met criteria for an IM and for SSc at 12 and 36 months from ILD diagnosis, respectively. In addition, 2 patients from the IPAF group developed ARS criteria over time, but still did not meet criteria for IM or another CTD, hence keeping their initial classification.

### Distribution of interstitial pneumonia with autoimmune features defining items in the diagnostic subgroups

Characterization of the process according to the IPAF defining variables is summarized in Supplementary File 4. As regards to clinical features, inflammatory arthralgia / arthritis (49%) and Raynaud’s phenomenon (20% of cases) were relatively prevalent in the IPAF group, while in contrast, SSc highly specific traits -such as sclerodactyly or digital scars- were only found in patients diagnosed with CTD-IP.

With respect to immunological markers (Supplementary File 4), RF and ANA could be found in the 3 groups, with titers not differing significantly between them. Considering ANA, the presence of anti-Ro60, low titers of anti-dsDNA antibodies and Hep2 indirect immunofluorescence (IFA) nucleolar patterns was observed within the uAIP subgroup, together accounting for a 50% of cases fulfilling the serological IPAF domain. In contrast, all myositis specific antibodies (MSA) were allocated to either of CTD or IPAF subgroups, while SSc-specific autoantibodies were exclusively described in patients fulfilling SSc classification criteria. Finally, ACPA were only associated to CTD-IP or IPAF cases, albeit the latter had significantly lower titers (*p* < 0.001).

Remarkably, there was a predominance of UIP/possible UIP patterns in our cohort (42% of cases) as compared to 33% of non-specific interstitial pneumonia (NSIP) patterns, which were second in frequency. The distribution of radiographic patterns between diagnostic groups did not reach significant differences. Since UIP does not score as an IPAF morphological trait, additional requirements were needed in order to assign these patients to the IPAF subgroup. The value of thoracic multi-compartment involvement or signs was reflected by their appearance in 27 and 45% of patients from the CTD and the IPAF subgroups, respectively. In particular, there were 13 patients diagnosed with IPAF who did not fulfil the clinical domain (Supplementary File 5). Interestingly, 4 of them with an UIP/possible UIP pattern and 2 other with an unclassifiable IP pattern could meet IPAF criteria due to the presence of thoracic multi-compartment involvement or signs in the CT scans. As shown in Supplementary File 5, there were 2 further cases who did not meet the radiographic domain either, but were classified after performance of a surgical lung biopsy. These consisted of a patient with a possible UIP pattern plus high RF titer and another one with an unclassifiable radiographic pattern and anti-Ro52 antibodies.

### Nailfold videocapillaroscopy

**Figure 1:**
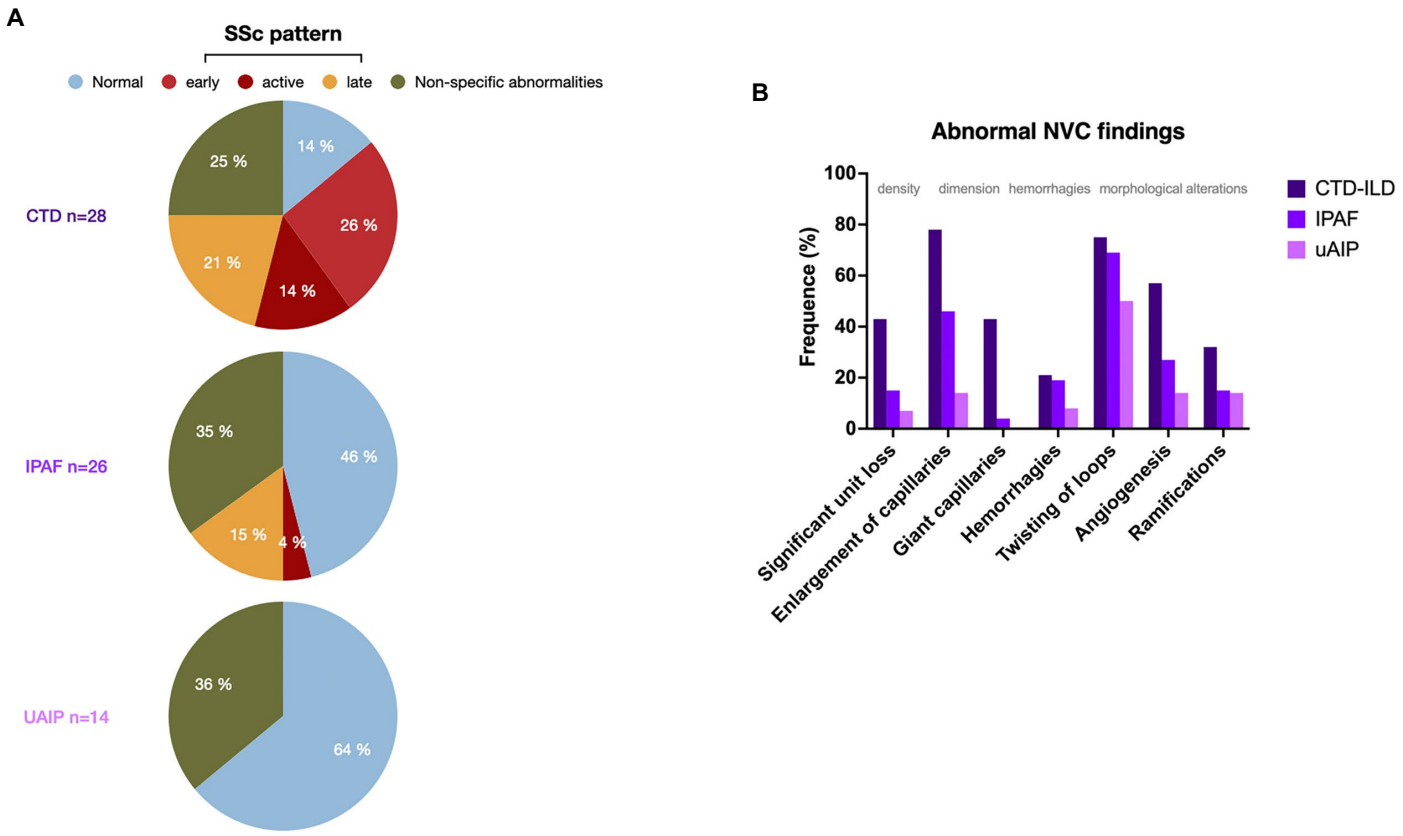
**(A)** Patterns of nailfold videocapillaroscopy (NVC) in the three diagnostic groups shown as percentage of the studies performed. **(B)** Distribution of nailfold videocapillaroscopy (NVC) abnormalities in the patients from the 3 diagnostic groups shown as percentage of the studies performed. SSc: systemic sclerosis. CTD: connective tissue disease, IPAF: interstitial pneumonia with autoimmune features. uAIP: undifferenciated autoimmune interstitial pneumonia. ILD: interstitial lung disease.

An nailfold videocapillaroscopy (NVC) had been performed as part of the workup in 68 of our patients (58%) of whom 26 were diagnosed with IPAF and 14 with uAIP. Of note, abnormal NVC findings were found in 14 (54%) and 5 (36%) of the patients from these two groups, respectively (Figure 1A). An SSc-specific pattern was observed in 5 (19%) of the IPAF patients, but in none with uAIP. Abnormal capillary shapes, such as twisting, ramifications and angiogenesis, were observed in patients from the 2 diagnostic subgroups (Figure 1B). Interestingly, 25 out of the 43 patients with NVC abnormalities did not have Raynaud’s phenomenon and some of them even fail to display any other digital lesions (Supplementary File 6). Subsequently, we analyzed which of the process-related variables along with Raynaud’s phenomenon could better predict the presence of abnormalities at NVC. As detailed in Table 3, both the presence of telangiectasia (p 0.023) and an NSIP radiographic pattern (p 0.041) were associated to abnormal NVC findings. An SSc pattern was found in all patients with digital ulcers (p 0.049), whereas the presence of giant capillaries was strongly associated to telangiectasia (p 0.004), digital ulcers (p 0.035), Raynaud’s phenomenon (p 0.027) and puffy fingers (p 0.017). Intriguingly, of all autoantibodies, anti-Ro predicted capillary enlargement (p 0.027), giant capillaries (p 0.024) and angiogenesis (p 0.046), while the presence of ramifications could be predicted by an ARS diagnosis (p 0.024) or an NSIP pattern (p 0.012).

**Table 3:** Predictors of nailfold videocapillaroscopy (NVC) major alterations.

	Telangiectasia	Digital ulcers	Raynaud’sphenomenon	Puffyfingers	Ro	NSIP	ARS
OVERALL PATTERN							
*SScpattern*		100% PPV; 56% NPV; *p* 0.049					
ABNORMAL NVC FINDINGS	PPV 100%; NPV 42%; *p* 0.023					OR 2.9 [1; 8.3] *p* 0.041	
*Capillarydimension*							
*Enlargement of capillaries*	PPV 100%; NPV 53%; *p* 0.006	100% PPV; NPV 50%; p 0.057	OR 3.73 [1.2; 11.3] *p* 0.02		OR 3.21 [1.13; 9.1] *p* 0.027		
*Giantcapillaries*	OR 10.6 [2.1; 53.3] *p* 0.004	OR 7.8 [1.1; 52.8] p 0.035	OR 4.16 [1.2; 14.7] *p* 0.027	OR 7.56 [1.4; 39.6] *p* 0.017	OR 4.5 [1.2; 16.6] p 0.024		
*Hemorrhages*	OR 6.37 [1.3; 30.7] *p* 0.021						
*CapillaryShapes*							
*Angiogenesis*					OR 2.84 [1.0; 7.9] p 0.046		
*Ramifications*						OR 5.9 [1.5; 23.5] p 0.012	OR 4.18 [1.2; 14.5] p 0.024

NSIP: non-specific interstitial pneumonia. ARS: anti synthetase syndrome. PPV: positive predictive value. NPV: negative predictive value. OR: odds ratio. Square brackets show 95% confidence intervals.

## Discussion

We present data from a retrospective cohort of similar demographics to other reported IIP populations (19, 20). Also alike many working groups, there was a considerable number of patients diagnosed with undifferentiated or pulmonary-dominant CTD before the concept of IPAF was introduced. In our patients, IPAF criteria showed a good performance in capturing former undifferentiated ILD cases. Up to 71% of the patients without an overt CTD could be classified using IPAF definition, which is remarkable considering that classification criteria of most CTD have been updated in the last few years in order to include patients at early stages of the disease.

However, the IPAF subgroup possible comprises distinct disease subtypes. Seeking to stratify patients according to more homogeneous criteria, we further explored distribution of the individual IPAF-defining items in the whole cohort. The fact that SSc-specific autoantibodies as well as digital lesions, such as sclerodactyly and scars, were limited to the subgroup of CTS-IP patients, not only supports the accuracy of the current SSc classification criteria, but also indicates that these traits might add little value to the identification of IPAF cases. Conversely, an important amount of IPAF patients overlapped with the ARS syndrome, either by sharing a defining antibody or typically-associated clinical features. This fact has been observed already, as well as the tendency of IPAF with ARS-related manifestations or antibodies to evolve to a definite CTD over time (21–23). In this regard, the need to consider segregation of ARS-like cases from other IPAF patients has been recently put forward (24, 25). Indeed, ARS antibodies have shown an invaluable role both in defining CTD overlapping phenotypes and in forecasting prognosis (26, 27). However, the fact that only anti-Jo1 antibodies are included as serological marker in the latest IM classification criteria leaves a diagnostic gap for those patients with non-anti-Jo1 ARS antibodies, which is currently being filled with the IPAF definition as also with preliminary sets of classification criteria for ARS syndrome (28). In order to bypass the gnosologic conundrum it is tempting to consider the presence of these highly specific antibodies as definitory of an underlying CTD in subjects with ILD, even in the absence of other manifestations. This would be of particular interest in those patients with ARS antibodies who present with a pulmonary-dominant disease and an UIP-like radiographic pattern, as has been described (29). This situation can be visualized in 1 patient from our IPAF cohort with anti-Jo1 who underwent a diagnostic surgical lung biopsy. Also to highlight is the presence of anti-Ha and anti-KS antibodies in 2 patients from our IPAF subgroup with pulmonary-dominant forms of ILD. Some years ago, these patients would have been difficult to characterize since commercial detection kits for these antibodies were not available (30). This suggests that patients with an autoimmune IP could remain uncaptured with current IPAF criteria. Along the same line, the group of Karolinska has uncovered the presence of serum immune-reactivity toward practically all aminoacyl tRNA synthetases in patients with IM. Although the relevance of these findings is yet to be clarified, it appears that closing the serological gap of the ARS syndrome could increase sensitivity for IPAF diagnosis (31).

In contrast with MSA and SSc-specific antibodies, the interpretation of positive RF, anti-Ro antibodies or low titers of anti-dsDNA and ACPA in patients with a pulmonary-dominant condition is challenging and performance of a surgical lung biopsy at the beginning of the disease -particularly in patients showing UIP radiographic patterns–might be a good choice both to correctly characterize the disease but also to better understand pathophysiological processes involved.

In this study, we have postulated that the assessment of digital microvascular pathology with NVC would increase sensitivity for the detection of IPAF, since the presence of NVC abnormalities is considered highly indicative of an undelying CTD. Particularly, the presence of giant capillaries or the combination of significant capillary loss and abnormal shapes predicts the development of SSc in patients with Raynaud’s phenomenon or with SSc-specific autoantibodies. Moreover, severity of the NVC lesions in patients with SSc might herald the development of SSc-IP, as it has been recently pointed out (32). Indeed, NVC is being increasingly used in the characterization of ILD patients and a clinical suspicion of an underlying CTD. A recently published metaanalysis concluded that the “late SSc” NVC pattern could associate to ILD not only in the context of CTD but also in patients diagnosed with IPAF or IIP (9). Nonetheless, formal studies approaching the relationship between qualitative NVC findings and the different ILD phenotypes are necessary (9, 32). Our study provides relevant information in this respect. We underscore that many patients without Raynaud’s phenomenon showed NVC abnormalities, including some who displayed an SSc pattern. Our data are in agreement with previous descriptions in patients with ARS (33) in whom an association with capillary ramifications was observed. As expected, digital macroscopic alterations within the clinical domain of the IPAF definition were found to associate with NVC abnormalities. However, as illustrated in our study, these alterations may be subtle or even absent and still the patients can have NVC abnormalities. As regards to other disease-related features, an NSIP pattern or the presence of anti-Ro antibodies were also associated to NVC alterations, findings which further support the potential role of this exam in the classification of unclear ILD cases. In our cohort, addition of NVC abnormalities to IPAF clinical criteria would have allowed classification of 4 of our uAIP patients, who had an NSIP (or a mixed NSIP-OP) pattern and an additional one with an UIP pattern, no extra-pulmonary clinical findings and anti-Ro antibodies. Nonetheless, in contrast with IPAF cases, none of the uAIP patients displayed an SSc specific NVC pattern, but non-specific abnormalities, which are less defined and potentially subject to the examiner interpretation. This fact highlights the need of expert consensus in this field (34).

In summary, we provide a thorough description of the characteristics of patients with autoimmune IP from a real-world cohort, illustrating that the assessment of the individual IPAF defining items can help identify homogeneous subgroups of the disease beyond the diagnostic classification. This segregation is necessary in order to advance to a precision-based medicine in this complex field (35). In addition, while some clinical features appear to be under-represented in patients without an overt underlying CTD, performance of NVC should be encouraged since it might help improve current IPAF definition and further distinguish 2 types of disease according to the presence or absence of autoimmune vasculopathy.

## Data availability statement

The original contributions presented in the study are included in the article/Supplementary material, further inquiries can be directed to the corresponding author.

## Ethics statement

The studies involving human participants were reviewed and approved by The HU Fundación Jiménez Díaz-IIS Ethics Committee. Written informed consent for participation was not required for this study in accordance with the national legislation and the institutional requirements.

## Author contributions

FR-B: contributed to the conception of the work, data acquisition and interpretation and manuscript writing. MR-N: contributed to the conception of the work, definition of variables, interpretation of data and revising the manuscript. CM: contributed to data acquisition and revising the manuscript. LE: contributed to data analysis and manuscript writing. MM-B: contributed to data acquisition and revised the manuscript. MS: contributed to data acquisition and revised the manuscript. OD: contributed to data acquisition and interpretation and revised the manuscript. OS-P: contributed to the conception of the work and the data acquisition, analysis and interpretation, and manuscript writing. All authors contributed to the article and approved the submitted version.

## Funding

This study was supported by a grant from the Carlos III Institute of Health, Ministry of Science and Innovation (ISCIII, AES PI20/00250), and FEDER funding.

## Conflict of interest

The authors declare that the research was conducted in the absence of any commercial or financial relationships that could be construed as a potential conflict of interest.

## Publisher’s note

All claims expressed in this article are solely those of the authors and do not necessarily represent those of their affiliated organizations, or those of the publisher, the editors and the reviewers. Any product that may be evaluated in this article, or claim that may be made by its manufacturer, is not guaranteed or endorsed by the publisher.
