# Supplementary figures and images for: Fine-tuning characterization of patients with interstitial pneumonia and an underlying autoimmune disease in real-world practice: We get closer with Nailfold videocapillaroscopy
Source: Front Med (Lausanne). 2023 Feb 15;10:1057643. doi: 10.3389/fmed.2023.1057643 (PMC9975591; doi:10.3389/fmed.2023.1057643)

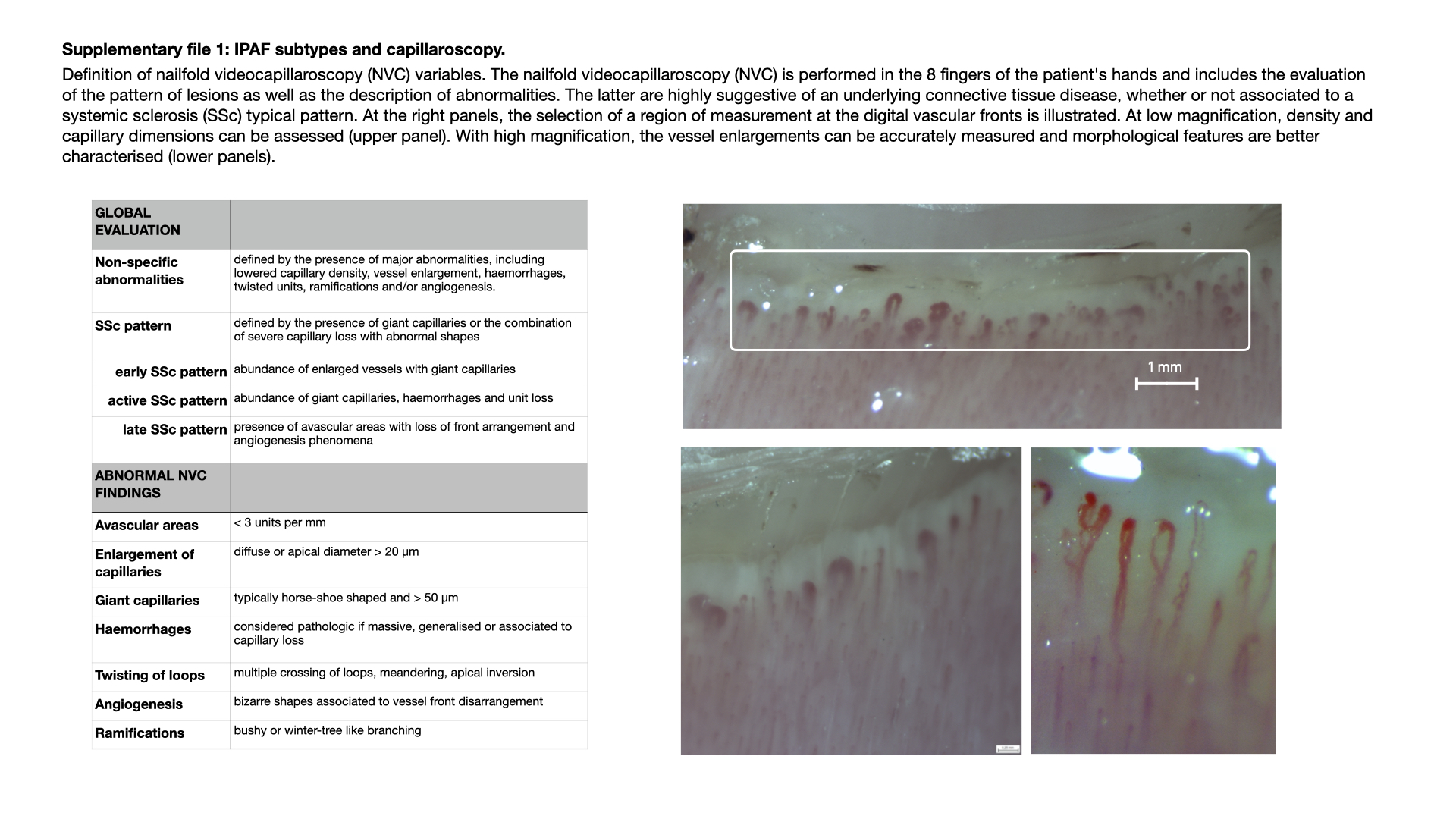

Supplement: Supplementary file 1 [file Image_1.jpeg]

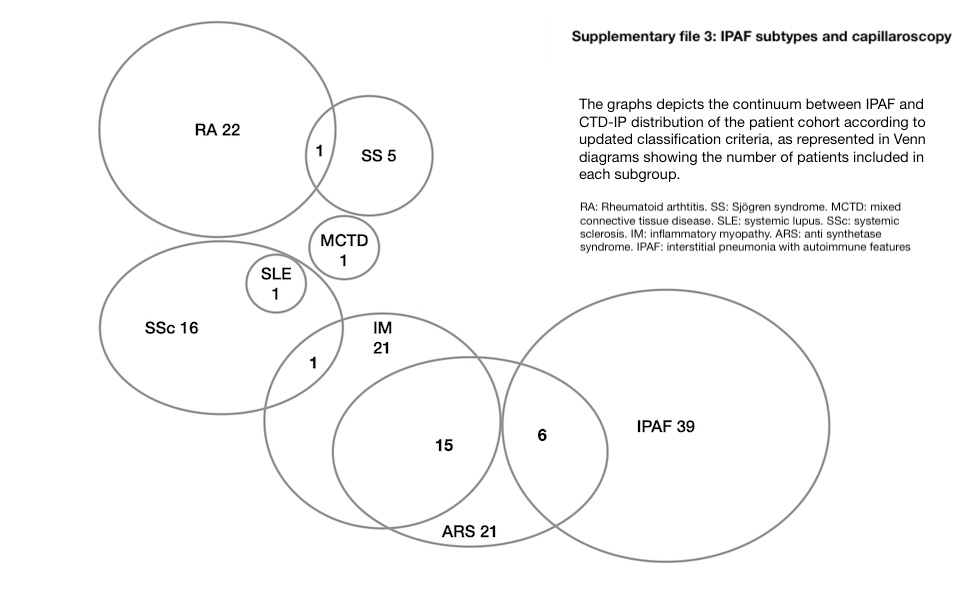

Supplement: Supplementary file 3 [file Image_2.png]
