# Supplementary material for: Fine-tuning characterization of patients with interstitial pneumonia and an underlying autoimmune disease in real-world practice: We get closer with Nailfold videocapillaroscopy
Source: Front Med (Lausanne). 2023 Feb 15;10:1057643. doi: 10.3389/fmed.2023.1057643 (PMC9975591; doi:10.3389/fmed.2023.1057643)
Supplement: Supplementary file 2 [file Data_Sheet_1.PDF]

**Supplementary file 2:** IPAF subtypes and capillaroscopy.

ARS subgroup. Solomon's criteria were used to assign patients to the anti synthetase syndrome (ARS) subgroup.

| | **ARS** |
|---|---|
| **Number** | 21 |
| **Women, n (%)** | 16 (76) |
| **Ever smoker, n (%)** | 14 (67) |
| **Smoking status, n: never, past, active smoker** | 7, 8, 6 |
| **Age at onset, mean (SEM) *median*** | 53.2 (3.3) *56* |
| **Age at endpoints, mean (SEM) *median*** | 63.5 (2.6) *64* |
| **Disease duration, mean (SEM) *median*** | 8.2 (1.1) *9* |
| **Follow-up, (patient-years)** | 170 |
| **Respiratory onset, n** | 7 |
| **Extra-respiratory onset, n** | 6 |
| **Synchronic pulmonary and extra pulmonary disease at onset, n** | 8 |
