# Supplementary material for: Fine-tuning characterization of patients with interstitial pneumonia and an underlying autoimmune disease in real-world practice: We get closer with Nailfold videocapillaroscopy
Source: Front Med (Lausanne). 2023 Feb 15;10:1057643. doi: 10.3389/fmed.2023.1057643 (PMC9975591; doi:10.3389/fmed.2023.1057643)
Supplement: Supplementary file 4 [file Data_Sheet_2.PDF]

Supplementary file 4: IPAF subtypes and capillaroscopy

Distribution of clinical, radiographic and serological variables according to the IPAF definition in each of the diagnostic subgroups. NSIP: non-specific interstitial pneumonia. OP: organized pneumonia. LIP: lymphoid interstitial pneumonia. UIP: usual interstitial pneumonia.MSA: myositis specific autoantibodies. ARS ab: antibodies against anti aminoacyl tRNA synthetases. SSc: systemic sclerosis. ATA: anti topoisomerase I antibodies. ARA: anti RNA polymerase III antibodies. ACA: anti centromere antibodies.

	Total cohort	CTD-ILD (n = 63)	IPAF (n = 39)	uAIP (n = 16)	Pearson Chi2
**Clinical features, n (%)**					
**Arthritis, morning stiffness, synovitis**	64 (54)	44 (70)	19 (49)	1 (6)	p < 0.001
**Puffy fingers**	11 (9)	8 (13)	3 (8)	0	ns
**Sclerodactyly**	5 (4)	5 (8)	0	0	ns
**Digital ulcers**	8 (7)	7 (10)	1	0	ns
**Digital scars**	2 (2)	2 (3)	0	0	ns
**Telangiectasia**	10 (8)	7 (10)	3 (8)	0	ns
**Mechanic's hands**	16 (14)	12 (19)	4 (10)	0	ns
**Raynaud's phenomenon**	34 (29)	26 (41)	8 (20)	0	p 0.002
**Radiographic patterns, n (%)**					
**NSIP**	41 (33)	25 (40)	12 (31)	4 (25)	ns
**OP**	4 (3)	1	3 (8)	0	ns
**mixed NSIP-OP**	3 (2)	1	1	1	
**LIP**	1	0	1	0	
**UIP / possible UIP**	51 (42)	24 (38)	17 (44)	9 (56)	ns
**unclassifiable**	22 (19)	12 (19)	5 (13)	2 (13)	ns
**Thoracic multicompartimental signs, n (%)**					
**any**	34 (30)	16 (27)	18 (46)	0	p 0.002
**pleural effusion/thickening**	11 (9)	5 (8)	6 (15)	0	ns
**pericardial effusion/thickening**	10 (8)	5 (8)	5 (13)	0	ns
**airway alteration**	2 (2)	0	2 (5)	0	
**vasculopathy**	19 (16)	8 (13)	11 (28)	0	p 0.024
**Autoantibodies**					**Pearson Chi2/ ANOVA**
**Rheumatoid factor (RF), n (%)**	44 (37)	27 (43)	15 (38)	2 (13)	ns
**RF (+ criterium), n (%)**	37 (31)	23 (36.5)	12 (31)	2 (13)	ns
**RF titer IU/ml, mean (SEM) *median***	357 (88)	338 (123) *173*	337 (138) *179*	88 (21) *88*	ns
**Anti citrullinated peptide antibodies (ACPA) IU/ml, n (%)**	26 (22)	21 (34)	5 (12.5)	0	p 0.004
**ACPA titer, mean (SEM) *median***	226 (30)	262 (32) *304*	69 (22) *42*	0	p < 0.001
**Anti nuclear antibodies (ANA), n (%)**	85 (73)	42 (67)	30 (77)	13 (81)	ns
**ANA (+ criteria), n (%)**	76 (64)	44 (70)	25 (64)	7 (44)	ns
**ANA titer, mean (SEM) *median***	408 (49) *320*	428 (79) *320*	411 (72) *320*	327 (108) *160*	ns
**Anti dsDNA, n (%)**	7 (6)	2 (3)	4 (10)	1 (6)	ns
**MSA, n (%)**	28 (24)	20 (33)	8 (20)	0	ns
**ARS ab, n (%)**	23 (20)	15 (25)	8 (20)	0	ns
**Jo1, n (%)**	10 (9)	8 (13)	2 (5)	0	ns
**SSC specific ab, n (%)**	8 (7)	8 (13)	0	0	ns
**ATA, n (%)**	6 (5)	6 (10)	0	0	
**ARA, n (%)**	1	1	0	0	
**ACA, n (%)**	1	1	0	0	
**Ro, n (%)**	39 (33)	27 (43)	10 (26)	2 (13)	p 0.029
**Ro52, n (%)**	18 (17)	13 (21)	5 (13)	0	p 0.044
**Ro60, n (%)**	13 (12)	6 (10)	5 (13)	2 (13)	ns
