# Supplementary material for: Fine-tuning characterization of patients with interstitial pneumonia and an underlying autoimmune disease in real-world practice: We get closer with Nailfold videocapillaroscopy
Source: Front Med (Lausanne). 2023 Feb 15;10:1057643. doi: 10.3389/fmed.2023.1057643 (PMC9975591; doi:10.3389/fmed.2023.1057643)
Supplement: Supplementary file 5 [file Data_Sheet_3.PDF]

Supplementary file 5: IPAF subtypes and capillaroscopy

Disease characteristics of patients classified as interstitial pneumonia with autoimmune features (IPAF). The variables scoring for classification are highlighted (white boxes). ANA: antinuclear antibodies; IF: immunofluorescence; MSA: myositis specific antibodies; SSc: systemic sclerosis; RF: rheumatoid factor; ACPA: anti citrullinated peptide antibodies; CT: computed tomography; NVC nailfold videocapillaroscopy; UIP: usual interstitial pneumonia; NSIP: non-specific interstitial pneumonia; OP: obliterative pneumonia; LIN: lymphoid interstitial pneumonia

arthralgia / synovitis	puffy fingers	sclerodactyly	digital ulcers	digital pitting scars	telangiectasia	mechanic's hands	Raynaud's phenomenon	ANA titer (1:)	ANA IF pattern	dsDNA	MSA	Ro	Ro60	Ro52	PmScl	SSc-specific antibodies	RF titer	ACPA	CT pattern	pleural effusion/ enlargement	pericardial effusion/ enlargement	small airway disease	pulmonary vasculopathy	histopathology (surgical lung biopsy)	NVC major pathology
No	Yes	No	No	No	No	No	Yes	320	homogeneous	No	No	Yes	No	Yes	No	No		No	UIP	No	No	No	Yes		No
Yes	No	No	No	No	No	No	No	320	nucleolar	No	No	No	No	No	No	No		No	UIP	No	No	No	No		
No	No	No	No	No	No	No	No	80	mixed	No	No	Yes	No	Yes	No	No		No	NSIP	No	No	No	No	lymphoid aggregates with germinal centres	Yes
Yes	No	No	No	No	Yes	No	Yes			No	No	No	No	No	No	No	700	Yes	UIP	No	No	No	No	other	
Yes	No	No	No	No	No	No	No			No	No	No	No	No	No	No	247	Yes	OP	No	No	No	No		No
No	No	No	No	No	Yes	No	No	640	mixed	No	No	Yes	Yes	No	No	No		No	possible UIP	No	No	No	No		Yes
Yes	No	No	No	No	Yes	Yes	No	1.280	nucleolar	No	No	No	No	No	No	No		No	non-classifiable	Yes	Yes	No	No	non-classifiable	Yes
Yes	No	No	No	No	No	No	No	160	fine speckled with multiple dots	No	No	No	No	No	No	No		No	LIN	No	No	No	No	lymphoid aggregates with germinal centres	Yes
No	No	No	No	No	No	No	No			No	No	No	No	No	No	No	279	No	possible UIP	No	No	No	Yes		
No	Yes	No	No	No	No	No	No			No	No	No	No	No	No	No	40	No	UIP	Yes	No	No	No		
Yes	No	No	No	No	No	No	Yes	320	unspecific	No	PL7	No	No	No	No	No		No	UIP	No	No	No	Yes		Yes
Yes	No	No	No	No	No	Yes	Yes			No	PL7	No	No	No	No	No		No	NSIP	No	Yes	No	No		Yes
No	No	No	No	No	No	No	No	160	homogeneous	No	No	No	No	No	No	No	41	No	possible UIP	No	No	No	Yes		Yes
Yes	No	No	No	No	No	No	No	160	unspecific	No	No	No	No	No	No	No	179	No	non-classifiable	No	No	No	No		No
No	No	No	No	No	No	No	No	640	fine speckled	No	No	Yes		No	No	No		No	UIP	Yes	No	No	Yes		No
Yes	No	No	No	No	No	No	No	640	fine speckled	No	No	Yes	Yes	No	No	No		Yes	UIP	No	No	No	Yes		Yes
Yes	No	No	No	No	No	No	No	320	unspecific	No	No	No	No	No	No	No		No	non-classifiable	No	No	No	No		Yes
No	No	No	No	No	No	No	No	80	unspecific	Yes	No	No	No	No	No	No	18	No	non-classifiable	Yes	No	No	No		
No	Yes	No	No	No	No	No	Yes			No	PL12	No	No	No	No	No		No	NSIP	No	No	No	No		No
Yes	No	No	No	No	No	No	No	80		No	No	No	No	No	No	No	35	No	NSIP	No	No	No	No		
Yes	No	No	No	No	No	No	No	1.280	nucleolar	No	No	No	No	No	Yes	No	20	No	NSIP	No	No	No	No		Yes
No	No	No	No	No	No	No	No			Yes	No	No	No	No	No	No		No	NSIP	No	No	No	Yes		Yes
No	No	No	No	No	No	No	No	320	unspecific	No	No	No	No	No	No	No		No	UIP	No	No	No	Yes		
Yes	No	No	No	No	No	Yes	No	80		No	Jo1	Yes	Yes	Yes	No	No		No	NSIP	No	No	No	No	OP	No
Yes	No	No	No	No	No	No	No	1.280	homogeneous	Yes	No	No	No	No	No	No		No	NSIP	No	No	No	No		No
Yes	No	No	No	No	No	No	No	640	fine speckled	No	No	No	No	No	No	No	516	No	NSIP	Yes	No	No	No		
Yes	No	No	No	No	No	Yes	No	160	cytoplasmic	No	PL7	No	No	No	No	No		No	NSIP	No	No	No	No		No
No	No	No	Yes	No	No	No	Yes	160	fine speckled	No	No	No	No	No	No	No		No	NSIP	Yes	Yes	Yes	Yes		No
Yes	No	No	No	No	No	No	No		fine speckled	No	No	No	No	No	No	No	205	No	UIP	No	No	No	No		
No	No	No	No	No	No	No	No	1.280	homogeneous	No	No	Yes	No	Yes	No	No		No	non-classifiable	No	No	No	No	diffuse lymphoplasmocytic infiltration	No
No	No	No	No	No	No	No	No	320	fine speckled	Yes	No	Yes	Yes	No	No	No		No	OP	No	No	No	No		
Yes	No	No	No	No	No	No	No			No	No	No	No	No	No	No	142	Yes	possible UIP	No	No	No	No		
No	No	No	No	No	No	No	No	640	nucleolar	No	Jo1	No	No	No	No	No		No	UIP	No	No	No	Yes	OP	No
No	No	No	No	No	No	No	No	320	cytoplasmic	No	KS	No	No	No	No	No		No	NSIP	No	Yes	No	No	OP	Yes
No	No	No	No	No	No	No	Yes			No	No	No	No	No	No	No		Yes	OP	No	Yes	Yes	No	UIP	Yes
No	No	No	No	No	No	No	Yes	80	cytoplasmic	No	No	No	No	No	No	No	91	No	UIP	No	No	No	No		No
No	No	No	No	No	No	No	No	320	cytoplasmic	No	Ha	Yes	No	Yes	No	No		No	mixed NSIP-OP	No	No	No	Yes		Yes
Yes	No	No	No	No	No	No	No	80	fine speckled	No	No	Yes	Yes	No	No	No	420	No	possible UIP	No	No	No	No		
No	No	No	No	No	No	No	No	80	fine speckled	No	No	No	No	No	No	No	2.130	No	possible UIP	No	No	No	No	lymphoid aggregates with germinal centres	
