# Supplementary material for: Fine-tuning characterization of patients with interstitial pneumonia and an underlying autoimmune disease in real-world practice: We get closer with Nailfold videocapillaroscopy
Source: Front Med (Lausanne). 2023 Feb 15;10:1057643. doi: 10.3389/fmed.2023.1057643 (PMC9975591; doi:10.3389/fmed.2023.1057643)
Supplement: Supplementary file 6 [file Data_Sheet_4.PDF]

Supplementary file 6: IPAF subtypes and capillaroscopy

Cases with major pathology in nailfold videocapillaroscopy studies. The variables scoring for classification as interstitial pneumonia with autoimmune features (IPAF) are highlighted (white boxes). uAIP: undifferentiated autoimmune interstitial pneumonia; CTD: connective tissue disease; RF: rheumatoid factor; ACPA: anti citrullinated peptide antibodies; ANA: antinuclear antibodies; MSA: myositis specific antibodies; SSc: systemic sclerosis; CT: computed tomography; UIP:

Clinical subgroup	RF	ACPA	ANA titer (1:)	ANA pattern	dsDNA	MSA	SSc-specific antibodies	Ro	Ro60	Ro52	U1RNP	PmScl	CT pattern	pleural effusion/ enlargement	pericardial effusion/ enlargement	small airway disease	pulmonary vasculopathy	arthralgia / synovitls	puffy fingers	sclerodactyly	digital ulcers	digital pitting scars	tenalgiectasia	mechanic's hands	Raynaud's phenomenon
uAIP	No	No	160	homogeneous	No	No	No	No	No	No	No	No	NSIP	No	No	No	No	No	No	No	No	No	No	No	No
uAIP	No	No			No	No	No	No	No	No	No	No	NSIP	No	No	No	No	No	No	No	No	No	No	No	No
uAIP	No	No	80	unspecific	No	No	No	No	No	No	No	No	NSIP	No	No	No	No	No	No	No	No	No	No	No	No
uAIP	No	No	1.280	nucleolar	No	No	No	Yes	Yes	No	No	No	UIP	No	No	No	No	No	No	No	No	No	No	No	No
uAIP	No	No	80	homogeneous	No	No	No	No	No	No	No	No	NSIP-OP	No	No	No	No	No	No	No	No	No	No	No	No
IPAF	No	No	80	mixed	No	No	No	Yes	No	Yes	No	No	NSIP	No	No	No	No	No	No	No	No	No	No	No	No
IPAF	No	No	640	mixed	No	No	No	Yes	Yes	No	No	No	possible UIP	No	No	No	No	No	No	No	No	No	Yes	No	No
IPAF	No	No	1.280	nucleolar	No	No	No	No	No	No	No	No	non-classifiable	Yes	Yes	No	No	Yes	No	No	No	No	Yes	Yes	No
IPAF	No	No	160	fine speckled with multiple dots	No	No	No	No	No	No	No	No	LIN	No	No	No	No	Yes	No	No	No	No	No	No	No
IPAF	No	No	320	unspecific	No	PL7	No	No	No	No	No	No	UIP	No	No	No	Yes	Yes	No	No	No	No	No	No	Yes
IPAF	No	No			No	PL7	No	No	No	No	No	No	NSIP	No	Yes	No	No	Yes	No	No	No	No	No	Yes	Yes
IPAF	Yes	No	160	homogeneous	No	No	No	No	No	No	No	No	possible UIP	No	No	No	Yes	No	No	No	No	No	No	No	No
IPAF	No	Yes	640	fine speckled	No	No	No	Yes	Yes	No	No	No	UIP	No	No	No	Yes	Yes	No	No	No	No	No	No	No
IPAF	No	No	320	unspecific	No	No	No	No	No	No	No	No	non-classifiable	No	No	No	No	Yes	No	No	No	No	No	No	No
IPAF	Yes	No	1.280	nucleolar	No	No	No	No	No	No	No	Yes	NSIP	No	No	No	No	Yes	No	No	No	No	No	No	No
IPAF	No	No			Yes	No	No	No	No	No	No	No	NSIP	No	No	No	Yes	No	No	No	No	No	No	No	No
IPAF	No	No	320	cytoplasmic	No	KS	No	No	No	No	No	No	NSIP	No	Yes	No	No	No	No	No	No	No	No	No	No
IPAF	No	Yes			No	No	No	No	No	No	No	No	OP	No	Yes	Yes	No	No	No	No	No	No	No	No	Yes
IPAF	No	No	320	cytoplasmic	No	Ha	No	Yes	No	Yes	No	No	NSIP-OP	No	No	No	Yes	No	No	No	No	No	No	No	No
CTD	No	No	1.280		No	No	No	Yes			SÍ	No	NSIP	No	No	No	No	Yes	Yes	No	No	No	No	No	No
CTD	Yes	No	160	fine speckled	No	No	No	Yes	No	Yes	No	No	NSIP	No	Yes	No	No	No	Yes	No	No	No	No	No	Yes
CTD	No	No	640	mixed	No	No	RNApolIII	Yes			No	No	UIP	No	No	No	No	No	No	No	No	No	No	No	Yes
CTD	Yes	No	640	fine speckled with multiple dots	No	No	No	No	No	No	No	No	NSIP	No	No	No	No	No	Yes	No	No	No	Yes	No	Yes
CTD	Yes	No	320	nucleolar	Yes	No	No	Yes	Yes	Yes	No	No	possible UIP	No	No	No	No	No	No	Yes	Yes	No	No	Yes	Yes
CTD	No	No	320	unspecific	No	Jo1	No	Yes			No	No	possible UIP	No	Yes	No	Yes	Yes	No	No	No	No	No	No	No
CTD	No	No	160		No	Jo1	No	Yes	No	Yes	No	No	NSIP	No	No	No	No	Yes	No	No	No	No	No	Yes	No
CTD	No	No	640	nucleolar	No	No	Scl70	No	No	No	No	No	NSIP	No	No	No	No	No	No	Yes	No	No	Yes	No	Yes
CTD	No	No	160	fine speckled	No	Jo1	No	Yes	Yes	Yes	No	No	UIP	No	No	No	No	Yes	No	No	No	No	No	No	Yes
CTD	No	No	80	cytoplasmic	No	PL12	No	Yes	No	Yes	No	No	NSIP	No	No	No	No	Yes	No	No	No	No	No	Yes	Yes
CTD	No	No	80	fine speckled	No	MDA5	No	Yes	No	Yes	No	No	NSIP	No	No	No	No	Yes	No	No	No	No	No	No	No
CTD	No	No			No	No	Scl70	Yes			No	No	UIP	No	Yes	No	No	No	No	No	No	No	No	No	Yes
CTD	No	No	1.280	nucleolar	No	No	No	Yes	Yes	No	No	No	NSIP	No	No	No	No	Yes	No	Yes	Yes	Yes	Yes	No	Yes
CTD	No	No	640	fine speckled	No	No	Scl70	No	No	No	No	No	NSIP	No	No	No	No	Yes	No	No	No	No	No	No	Yes
CTD	No	No	80	fine speckled	No	Jo1	No	Yes			No	No	non-classifiable	No	No	No	Yes	Yes	No	No	No	No	No	Yes	No
CTD	Yes	No		cytoplasmic	No	EJ	No	No	No	No	No	No	NSIP	No	No	No	No	No	No	No	No	No	No	Yes	No
CTD	No	No			No	MDA5	No	No	No	No	No	No	NSIP	No	No	No	No	Yes	No	No	No	No	No	No	No
CTD	No	No	80	fine speckled	No	No	No	No	No	No	No	No	NSIP	No	No	No		No	No	No	No	Yes	No	No	Yes
CTD	No	No	320	mixed	No	No	No	No	No	No	No	No	NSIP	No	No	No		No	Yes	No	No	No	No	No	Yes
CTD	Yes	No	640		No	No	No	No	No	No	No	No	UIP	No	No	No	No	Yes	No	No	Yes	No	Yes	No	Yes
CTD	No	No	80	mixed	No	Jo1	No	Yes	No	Yes	No	No	NSIP	No	No	No	No	Yes	Yes	No	No	No	No	Yes	Yes
CTD	No	No	80	centromeric	No	PL12	CENP-B	Yes	No	Yes	No	No	non-classifiable	No	No	No	No	Yes	Yes	No	Yes	No	Yes	Yes	Yes
CTD	No	No			No	MDA5	No	Yes	No	Yes	No	No	non-classifiable	No	No	No	No	No	No	No	No	No	Yes	No	No
CTD	No	No	320	cytoplasmic	No	PL12	No	Yes	No	Yes	No	No	mixed NSIP-OP	No	No	No	No	No	No	No	No	No	No	Yes	No
